# ROS-scavenging hydrogel as protective carrier to regulate stem cells activity and promote osteointegration of 3D printed porous titanium prosthesis in osteoporosis

**DOI:** 10.3389/fbioe.2023.1103611

**Published:** 2023-01-17

**Authors:** Wenbin Ding, Qirong Zhou, Yifeng Lu, Qiang Wei, Hao Tang, Donghua Zhang, Zhixiao Liu, Guangchao Wang, Dajiang Wu

**Affiliations:** ^1^ Department of Orthopaedics, Changhai Hospital, Second Military Medical University, Shanghai, China; ^2^ Department of Histology and Embryology, College of Basic Medicine, Shanghai, China

**Keywords:** hydrogel, stem cells, 3D printed prosthesis, osteointegration, osteoporosis

## Abstract

Stem cell-based therapy has drawn attention as an alternative option for promoting prosthetic osteointegration in osteoporosis by virtue of its unique characteristics. However, estrogen deficiency is the main mechanism of postmenopausal osteoporosis. Estrogen, as an effective antioxidant, deficienncy also results in the accumulation of reactive oxygen species (ROS) in the body, affecting the osteogenic differentiation of stem cells and the bone formation i osteoporosis. In this study, we prepared a ROS-scavenging hydrogel by crosslinking of epigallocatechin-3-gallate (EGCG), 3-acrylamido phenylboronic acid (APBA) and acrylamide. The engineered hydrogel can scavenge ROS efficiently, enabling it to be a cell carrier of bone marrow-derived mesenchymal stem cells (BMSCs) to protect delivered cells from ROS-mediated death and osteogenesis inhibition, favorably enhancing the tissue repair potential of stem cells. Further *in vivo* investigations seriously demonstrated that this ROS-scavenging hydrogel encapsulated with BMSCs can prominently promote osteointegration of 3D printed microporous titanium alloy prosthesis in osteoporosis, including scavenging accumulated ROS, inducing macrophages to polarize toward M2 phenotype, suppressing inflammatory cytokines expression, and improving osteogenesis related markers (e.g., ALP, Runx-2, COL-1, BSP, OCN, and OPN). This work provides a novel strategy for conquering the challenge of transplanted stem cells cannot fully function in the impaired microenvironment, and enhancing prosthetic osteointegration in osteoporosis.

## Introduction

Osteoporosis is a systemic osteopathy characterized by decreased bone mass, destructed bone microstructure, and increased bone fragility, which is prone to fracture and has impaired bone regeneration and repair capacity (Aghebati-Maleki et al., 2019; Zhao et al., 2020). In bone homeostasis, the osteogenic differentiation of bone marrow mesenchymal stem cells (BMSCs) is an important issue in the repair of bone injuries, including fracture healing and osteointegration at the prosthesis interface after joint replacement (Wang et al., 2021). Once bone injuries occur, multiple signals trigger BMSCs in bone marrow or surrounding tissues to migrate to the injured regions and play a paracrine role (Chen et al., 2021; Du et al., 2021; Sheng et al., 2022). In addition, BMSCs also have a hand in the healing stages of inflammation, repair, and remodeling together with stromal cells, progenitor cells, pro-inflammatory (M1 phenotype) macrophages, and anti-inflammatory (M2 phenotype) macrophages (Zou et al., 2021; Arostegui et al., 2022). After directional differentiation into osteoblasts, BMSCs secrete a series of components of extracellular bone matrix to enhance the deposition of calcium ions *in situ*, thus inducing the formation of mature bone tissues (Grayson et al., 2015; Chen M. et al., 2022). Therefore, the quantity of BMSCs and their capacity to differentiate into osteoblasts in the injury microenvironment play a critical role in the early bone repair process.

However, the local osteogenic microenvironment of osteoporosis not only lacks BMSCs, but also has generally impaired osteogenic differentiation ability, which limits the repair of bone injury in osteoporosis (Liu et al., 2016; Li et al., 2022b). As a result, the osteointegration in osteoporosis after titanium alloy prosthesis implantation is often insufficient, which enhances the incidence of complications, e.g., prosthesis displacement and loosening, and periprosthetic fractures (Brandi, 2013; Bottai et al., 2015). Therefore, it is considered as an alternative strategy to induce bone repair and osseointegration by delivering exogenous BMSCs to the injured sites in osteoporosis (Hejazi et al., 2021; Laguna et al., 2022; Niu et al., 2022). Notably, Bai et al. prepared a functional hydrogel as the delivery carrier of BMSCs, and encapsulated the cells in the micropores of 3D-printed titanium alloy prosthesis, which significantly promoted the bone integration of prosthesis interface in the distal femur of osteoporotic rabbits (Bai et al., 2020c). This novel cell delivery strategy opens a new idea for the design of bioactive prosthesis that are conducive to osteointegration under osteoporosis. However, it needs to be emphasized that how the stem cells delivered to the target sites respond to the harsh conditions of disease microenvironment and fully play the role of paracrine and directional differentiation is the critical issue to tissue repair (Yu et al., 2021; Liu J. et al., 2022; Zhao et al., 2022). Specifically, in the local osteogenic microenvironment of osteoporosis, the lack of estrogen in postmenopausal osteoporosis will lead to the rapid accumulation of reactive oxygen species (ROS), including hydrogen peroxide (H_2_O_2_), hydroxyl radical (OH^−^), and superoxide anion ( $O_{2}^{-}$ ) in the bone marrow microenvironment, which is the main place for bone regeneration by offering multifarious biochemical, physical, and mechanical signals for various bone related cells (Qin et al., 2019; Schroeder, 2019; Wu et al., 2022). The increase level of ROS will inhibit the BMSCs differentiation and seriously impair the early osteointegration between implant-bone interfaces, thus resulting in increased risk of prosthesis related complications and secondary surgery after joint replacement (Lee et al., 2021; Zhang et al., 2021). Therefore, developing cell protective delivery carriers has great clinical significance for strengthening the osseointegration after prosthesis implantation.

**SCHEME 1:**
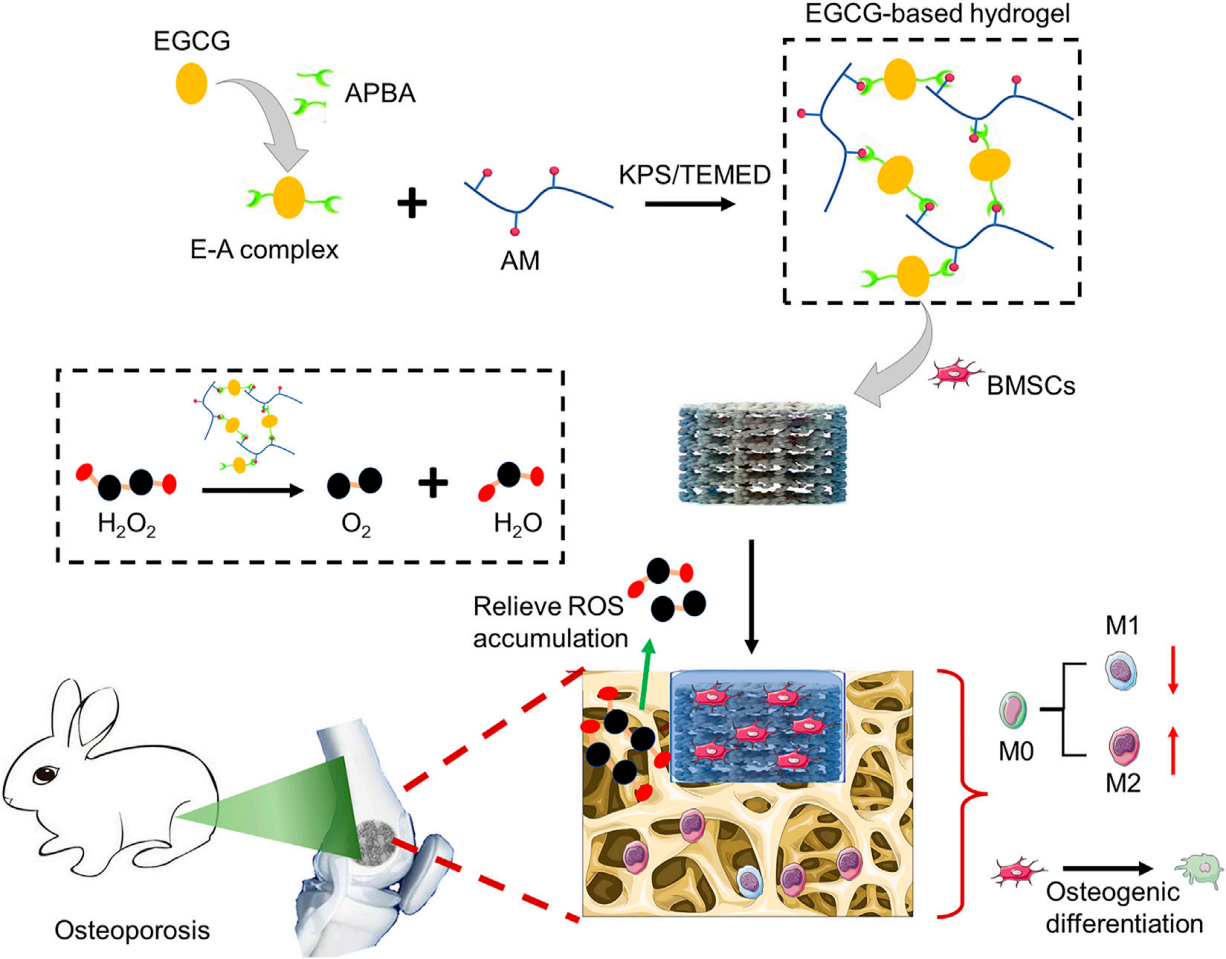
Schematic diagram of preparing ROS-scavenging hydrogel as protective carrier to regulate stem cells activity and promote osteointegration of 3D printed porous titanium prosthesis in osteoporosis.

To address this major challenge, researchers have made extensive efforts. At present, active ingredients (such as anti-inflammatory agents, exosomes, and nanoparticles) provide a feasible strategy for combating chronic inflammation caused by ROS accumulation and accelerating tissue regeneration (Zhao et al., 2021). Among them, natural compound, epigallocatechin-3-gallate (EGCG), the most abundant polyphenolic compound in green tea, has attracted considerable attention recently (Liu T. et al., 2022; Zhichao et al., 2022). EGCG has been widely studied and applied in biomedical field due to its superior physical and chemical properties, including anti-inflammatory, anti-oxidant, bactericidal, anti-aging, promoting angiogenesis, anti-cancer effects, and easy crosslinking to prepare hydrogels (Byun et al., 2021; Mi et al., 2021). Considering the accumulation of ROS in the osteoporotic microenvironment, herein, we prepared an EGCG-based hydrogel with ROS-scavenging ability as the cell delivery carrier to enhance the survival and osteogenic differentiation of BMSCs under oxidative stress, to improve the osseointegration of porous titanium alloy prosthesis in osteoporosis. Briefly, the resulting hydrogel was easily obtained by copolymerization of acrylamide (AM) with the E-A complex constructed by EGCG and 3-acrylamido phenylboronic acid (APBA). The substantial release of EGCG can scavenge the accumulated ROS and realize anti-oxidation, thus protecting the BMSCs encapsulated inside. As a protective cell carrier, hydrogel loaded with BMSCs is fully filled into the pores of 3D-printed porous titanium alloy prosthesis to construct a novel bioactive prosthesis interface to pave the way for osseointegration under osteoporosis (Scheme 1).

## Materials and methods

### Materials

Ti_6_Al_4_V powder was supplied by AK Medical (Beijing, China). EGCG, Cell Counting Kit-8 (CCK-8) assay, Calcein-AM/Propidium Iodide (PI) kit, and RIPA Lysis Buffer were provided by Beyotime Biotechnology (Shanghai, China). Ammonium persulphate (APS), AM, APBA, and N,N,N′,N′-Tetramethylethylenediamine (TEMED) were provided by Aladdin Bio-Chem Technology Co.,LTD. (Shanghai, China). Hydrogen peroxide (H_2_O_2_) aqueous solution (30%) was obtained from Beijing Chemical Works (Beijing, China). For cell culture, low glucose Dulbecco’s Modified Eagle’s Medium (LG-DMEM), fetal bovine serum (FBS), and streptomycin double antibody, and .25% trypsin EDTA were supplied by Gibco (Grand Island, NY, USA). Rabbit BMSCs (RBXMX-01001), osteogenic inducing differentiation medium of rabbit BMSCs, and alizarin red staining (ARS) were obtained from Cyagen Biosciences (Guangzhou, China). Phosphate buffer (PBS) and 4% paraformaldehyde were purchased from Solarbio (Beijing, China). Hematoxylin and eosin (H&E) stain was provided by Thermo Fisher Scientific (Shanghai, China). Eastep Super Total RNA Extraction Kit was purchased from Promega (Shanghai, China). Perfect Real Time RT reagent kit was provided by Takara Bio (Dalian, China). Enzyme linked immunosorbent assay (ELISA) kits were obtaind from Hengyuan (Shanghai, China). Primary antibodies, CD68, CD86, and CD163 were supplied by Abcam (Cambridge, UK). ROS probe 2,7-dichlorodihydrofluorescein diacetate (DCFH-DA) and fluorescent secondary antibodies were supplied by Sigma-Aldrich (St. Louis, MO, USA). The 4′,6-diamidino-2-phenylindole (DAPI) was purchased from Solarbio (Beijing, China).

### Hydrogel preparation

The E-A complex was prepared as previous report first (Zhao et al., 2021). Briefly, 92 mg EGCG was dissolved in 500 uL dimethylsulfoxide (DMSO) at a concentration of 184 mg/mL (.4 M). 76 mg APBA was add into 500 uL DMSO at a concentration of 152 mg/mL (.8 M). After adding the EGCG solution into the APBA solution, the mixture was mixed by a vortex mixer for 30 s and rest for 15 min to drive E-A complex formation. The prepared E-A complex (9 mM) was directly use to polymerize with AM (2 M) in the presence of TEMED and APS to obtain the resulting hydrogel at room temperature after purging with nitrogen for 5 min.

### Rheological tests of hydrogel

The rheological behaviors of the resulting hydrogel were evaluated by a TA rheometer (DHR-2). Specifically, the storage modulus (G′) and loss modulus (G″) were detected by placing the samples between parallel plates with the 20 mm diameter and the 1 mm gap. The experiments were carried out through a time sweep test at 37°C with a frequency of 10 rad^−1^ and 1% strain. In addition, strain amplitude sweep tests (*γ* = 1–1000%) were used to evaluate the critical strain value of the hydrogel at 37°C with 1 Hz frequency.

### Hydrogel degradation

For the *in vitro* degradation investigation, 100 mg freeze-dried hydrogels were weighted and placed in 5 mL PBS solutions (pH = 7.4) at 37°C and shaking at 100 rpm, respectively. At the predetermined time points, residual hydrogel samples were collected and weighed after freeze-drying. The mass remaining ratio of hydrogels was then calculated by the following formula: Mass remaining ratio (%) = W_t_/W_0_×100%, where the W_t_ is the weight of remaining hydrogels after degradation at predetermined time points and the W_0_ is initial weight of the hydrogels, respectively.

### EGCG release profile

The release profiles of EGCG in the obtained hydrogels were conducted in .01 M PBS at pH 7.4. In brief, 1 mL hydrogels were placed on the bottom of a 10 mL glass bottle, and added with 6 mL PBS to immerse the hydrogels. Then, the glass bottles were placed at 37°C and shaken at 100 rpm for different times (0 days, .5 days, 1 day, 2 days, 4 days, 6 days, 8 days, 10 days, 12 days, 14 days, 16 days, 18 days, and 20 days). At predetermined time intervals, 6 mL supernatants were collected and 6 mL fresh PBS was added. The release curves of EGCG were obtained according to Lambert-Beer’s Law on a Perkin-Elmer Lambda 950 UV-VIS spectrophotometer.

### Biocompatibility of hydrogel

The biocompatibility of the resulting hydrogels was evaluated by encapsulating 1×10^4^ rBMSCs in 100 μL hydrogel in 48-well plates. After incubation for 3 days, cell survival was evaluated by Calcein-AM/PI staining referencing the protocols of manufacturer. In simple terms, 100 μL calcein-AM solution was transferred into each well and incubated for 10 min, and then 100 μL PI solution was added and incubated for another 5 min in the dark at room temperature. After rinsing by PBS for 3 times, the stained cells were observed and photographed by the fluorescence microscope (IX53, Olympus, Japan), and the cell survival rates were further analyzed by an ImageJ software (NIH, Bethesda, MD, USA).

### Intracellular ROS scavenging

For detecting the ROS-scavenging capacity of hydrogel, rBMSCs (1×10^4^ cells/well) were encapsulated in 100 μL hydrogel or cultured with 100 μL PBS in 48-well plates for 48 h in LG-DMEM supplemented with 300 μM of H_2_O_2_, which are abbreviated as H_2_O_2_+PBS and H_2_O_2_+hydrogel group, respectively. For comparation, cells seeded in the normal medium without hydrogel and H_2_O_2_ as the normal control group (PBS group). Intracellular ROS levels were evaluated by an ROS probe (DCFH-DA). Briefly, the samples were rinsed by PBS for 3 times, and then 150 μL of DCFH-DA probe (20 μM) was added to each well and incubated for 20 min at room temperature. The fluorescence intensity indicating the intracellular ROS level was recorded by a fluorescence microscope and analyzed by ImageJ software. In addition, the H_2_O_2_ decomposing ability of hydrogel was further detected by the typical Ti(SO_4_)_2_ colorimetric method. Simply put, 1.33 mL of 24% Ti(SO_4_)_2_ and 8.33 mL of H_2_SO_4_ were dissolved in 50 mL of deionized water to form the Ti(SO_4_)_2_ solution. After the cells are incubated for 48 h under the above conditions, 100 µL of the supernatant from each sample was collected, and 200 µL of Ti(SO_4_)_2_ solution was added to the supernatant. The concentration of H_2_O_2_ could be determined by measuring the absorbance at 405 nm and calculating with the standard curve.

### Cell proliferation and survival under oxidative stress

Calcein-AM/PI staining and CCK-8 assay was used to detect the viability and proliferation of rBMSCs cultured in different substrates under oxidative stress. Briefly, cells were seeded on different substrates cultured with DMEM containing H_2_O_2_ in 48-well plates with an initiated density of 1×10^4^ cells/well. On the 4 day, the cell samples were labelled by Calcein-AM/PI kit to mark the cell survival, as described in the previous section. At the first, fourth and seventh day, the medium was discarded and the stem cells were rinsed by PBS for 3 times. Then, 300 μL of serum-free medium containing 10% CCK-8 solution was transferred to every well and incubated at 37°C for 2 h protecting from light. Finally, 100 μL of the solution was transferred to 96-well plates and the absorbance was detected at 450 nm by a microplate reader (Bio-Rad 680).

### Osteogenic differentiation under oxidative stress

Cells cultured in different substrates (1×10^4^ cells/well) in 48-well plates were incubated in an H_2_O_2_-supplemented osteogenesis inducing medium for 7 and 14 days. At the scheduled times, the samples were fixed in 4% paraformaldehyde and then stained with alizarin red dye (40 mM) for 20 min. The cell samples were rinsed gently with PBS for 3 times and observed by an optical microscope (MVX10, Olympus). To further quantitatively analyze the amount of calcium nodules deposited, 2% cetylpyridine chloride was used to dissolve the deposited calcium nodules for 30 min, and the absorbance was detected at 562 nm with a microplate reader.

In addition, quantitative reverse transcription polymerase chain reaction (RT-qPCR) was performed to evaluate the mRNA levels related to osteogenesis, including alkaline phosphatase (ALP), runt-related transcription factor 2 (Runx2), type I collagen (COL-1), bone sialoprotein (BSP), osteocalcin (OCN), and osteopontin (OPN). Briefly, the total RNA of rBMSCs grown on different substrates were collected using an RNA Extraction Kit according to the protocols. The first-strand cDNA was synthesized by reverse transcription of RNA using a PrimeScript RT reagent kit according to the manufacturer’s instructions. Finally, real-time PCR was done by a CFX Connect™ real-time PCR system (Bio-Rad) with an iTaq™ universal SYBR^®^ Green Supermix. The primer sequences are displayed in the Table 1. The relative mRNA expression levels were analyzed by the 2^−ΔΔCt^ method.

**TABLE 1:** Primers of target genes.

Target genes	Primers
	F: 5′- AGA​GTC​AGA​TTA​CAG​ATC​CCA​GG -3′
Runx-2	R: 5′- TGG​CTC​TTC​TTA​CTG​AGA​GAG​G -3′
	F:5′- GAA​CAG​AAC​TGA​TGT​GGA​ATA​CGA​A- 3′
ALP	R:5′- CAG​TGC​GGT​TCC​AGA​CAT​AGT​G - 3′
	F:5′- GAT​GTT​GAA​CTT​GTT​GTT​GCT​GAG​GG - 3′
COL-I	R:5′- GGC​AGG​CGA​GAT​GGC​TTA​TT - 3′
	F:5′- AAA​AGT​GAA​GGA​AAG​CGA​CGA​G - 3′
BSP	R:5′- CGT​GGA​GTT​GGT​GCT​GGT​G - 3′
	F:5′- GTG​ATT​TGC​TTT​TGC​CTG​TTT​G- 3′
OPN	R:5′- GGA​GAT​TCT​GCT​TCT​GAG​ATG​GG - 3′
	F:5′- GAA​CAG​ACA​AGT​CCC​ACA​CAG​C - 3′
OCN	R:5′- TCA​GCA​GAG​TGA​GCA​GAA​AGA​T - 3′
	F:5′- CTC​GTC​CCG​TAG​ACA​AAA​TGG​T - 3′
GAPDH	R:5′- GAG​GTC​AAT​GAA​GGG​GTC​GTT - 3′

### Preparation of 3D-Printed prosthesis and cell-loaded bioactive implants

The 3D-printed prosthesis were prepared by an Q10 Electron Beam Melting system (EBM, Arcam, Sweden) according to previous study (Li et al., 2018). The parameters of the cylindrical prosthesis were designed as diameter 6 mm, height 3 mm, porosity 70%, pore size 800 μm. In brief, pre-designed standard triangulation language (STL) file was delivered into the EBM system. And then the spherical Ti_6_Al_4_V powder was melted layer by layer according to the pre-designed parameters. All resulting porous titanium alloy prostheses were ultrasonically cleaned and sequentially washed in acetone, ethyl alcohol, and deionized water for 15 min at each producer. The actual parameters (e.g., porosity and pore size) were confirmed by micro-computed tomography system (Micro-CT, Skyscan1176, Bruker, Germany) and JSM-6700F scanning electron microscope (SEM), respectively.

To prepared the BMSCs-loaded bioactive prosthesis, the implants were placed in the moulds with a diameter of 7 mm, and then 1×10^6^ rBMSCs were mixed gently into 100 μL hydrogel solution and added into the moulds. The hydrogel fully filled into the pores of the prosthesis and gelled within 5 min.

### Establishment of osteoporosis model

A total of 40 female rabbits (5-month-old) were incorporated into the *in vivo* study after bilateral ovariectomy (OVX) or sham operation, as previous described (Bai et al., 2020a). In brief, the osteoporotic rabbit models were established by bilateral ovariectomy OVX under general anesthesia by 3% (w/v) pentobarbital at the dosage of 50 mg/kg. In brief, after skin preparation, the uterus and fallopian tubes were exposed through a median incision in the lower abdomen. The fallopian tube and its surrounding tissues were passively separated, the position of the ovary was found at the distal end of fallopian tube. The arteries and fallopian tubes were ligated, followed by bilateral OVX (*n* = 36). The abdominal cavity of rabbits in the Sham operation group (*n* = 4) were closed when we found the ovary without removing. Eight months after operation, four animals in OVX group and Sham group respectively were sacrificed. The serum estrogen level was analyzed by ELISA kit according to the manufacturer’s protocols, and the bone mass of distal femur was detected by a Micro-CT to prove the osteoporosis status.

### Prosthesis implantation

After the establishment of osteoporosis animal models, we implanted various prostheses in the left distal femur under sterile conditions. Briefly speaking, under general anesthesia by pentobarbital, a cylindrical defect with a diameter of 6 mm was made on the lateral condyle of the left distal femur of OVX rabbits by a surgical drill, and different porous titanium alloy prostheses were implanted into the bone defects. Finally, the incisions were sutured layer by layer with absorbable thread. Specific *in vivo* detections were divided into four groups, namely, pure porous titanium alloy prosthesis implantation (Ti group), porous titanium alloy prosthesis plus 1×10^6^ rBMSCs mixed in 100 μL PBS implantation (Ti@BMSCs group), pure hydrogel filled porous titanium alloy prosthesis implantation (Ti-HG group), and 1×10^6^ rBMSCs-loaded hydrogel filled porous titanium alloy prosthesis implantation (Ti-BMSCs@HG group), respectively.

### ROS-scavenging and macrophage polarization in osteoporosis model

After implantation for 2 weeks, four osteoporotic rabbits in each group were sacrificed and their left distal femurs were collected. After removal of titanium alloy prosthesis, the bone samples were fixed in 4% paraformaldehyde for 48 h and then decalcified by 12% ethylene diamine tetraacetic acid (EDTA) for 6 weeks. The samples were embedded in paraffin for routine histological operations to prepare about 4 μm thickness sections for immunofluorescence staining analysis. The accumulation and scavenging of ROS in bone tissue around the prosthesis were stained and observed by a ROS probe (DCHF-DA) according to the instructions of the kit. For immunofluorescence detections to observe macrophages polarization, sections were further incubated with CD68 plus CD86 and CD 68 plus CD163. In brief, the sections were rinsed by PBS gently and blocked with 3% BSA in PBS containing .2% Triton X-100 for 60 min. And then, the sections were incubated with the primary antibodies, including Runx-2 anti-mouse polyclonal antibody (1:200) plus OSX anti-rabbit monoclonal antibody (1:250), COL-1 anti-mouse polyclonal antibody (1:150) plus OCN anti-rabbit polyclonal antibody (1:200) overnight at 4°C. After rinsing by PBS for 3 times, the sections were incubated with 1:600 Cy3-conjugated goat anti-rabbit or 1:800 goat anti-mouse IgG DyLight 488-conjugated secondary antibodies for 1 h at 37°C. Finally, the nuclei were stained by DAPI (1:600) and the fluorescence images were observed by a fluorescence microscope and analyzed by ImageJ software. For histological analysis, we will select five visual fields of bone around the implant for analysis and calculate the average value as the parameter of this sample. At least three samples shall be included in each experimental group to obtain statistical results. In addition, the bone marrow blood around the prosthesis interfaces were collected to analyze the concentration of pro-inflammatory cytokines, such as tumor necrosis factor α (TNF-α), interleukin-1β (IL-1β), and IL-6 through ELISA kits according to the protocols.

### Micro-CT

Ten weeks after prosthesis implantation, the remaining osteoporotic animals were sacrificed and the left distal femurs were collected. Bone regeneration and osteointegration around prosthesis interface was scanned by a Micro-CT. The parameters during scanning are set to 90 kV voltage, 200 μA current, and 18 μm pixel size. The prosthesis contained cylindrical area was selected as the region of interest (ROI) for threshold segmentation to distinguish between bone and metal prosthesis by CTAn software, that is, white represented the scaffold and red represented the bone tissue. After 3D reconstruction, the quantitative morphometric analysis, including the ratio of bone volume/total volume (BV/TV, %), trabecular number (Tb.N, 1/mm), trabecular thickness (Tb.Th, μm), trabecular space (Tb.Sp, mm), and bone mineral density (BMD, g/cm^2^) were also analyzed by CTAn software.

### Histological evaluation

Ten weeks after prosthesis implantation, the bone samples of distal femur were collected, and dehydrated and embedded in methyl methacrylate. And then, the non-decalcified samples were sectioned into 150–300 μm thick, and followed by polishing to 40–50 μm by the transverse saw cuts and a polishing machine (Exact Apparatebau, Norderstedt, Germany). To observe the bone ingrowth into the porous implants, the sciles were stained with H&E dye based on the manufacturer’s protocols.

### Statistical analysis

All experimental detections were repeated at least 3 times independently. All of the data in current study was presented as means ± standard deviation, and analyzed with SPSS 19.0 (SPSS Inc., Chicago, IL, USA) *via* Student’s t-test and one-way analysis of variance (ANOVA). For inter group comparison, *p < .05, **p < .01, and ***p < .001 were considered statistically significant.

## Results and discussion

### Preparation and characterization of hydrogel

**FIGURE 1:**
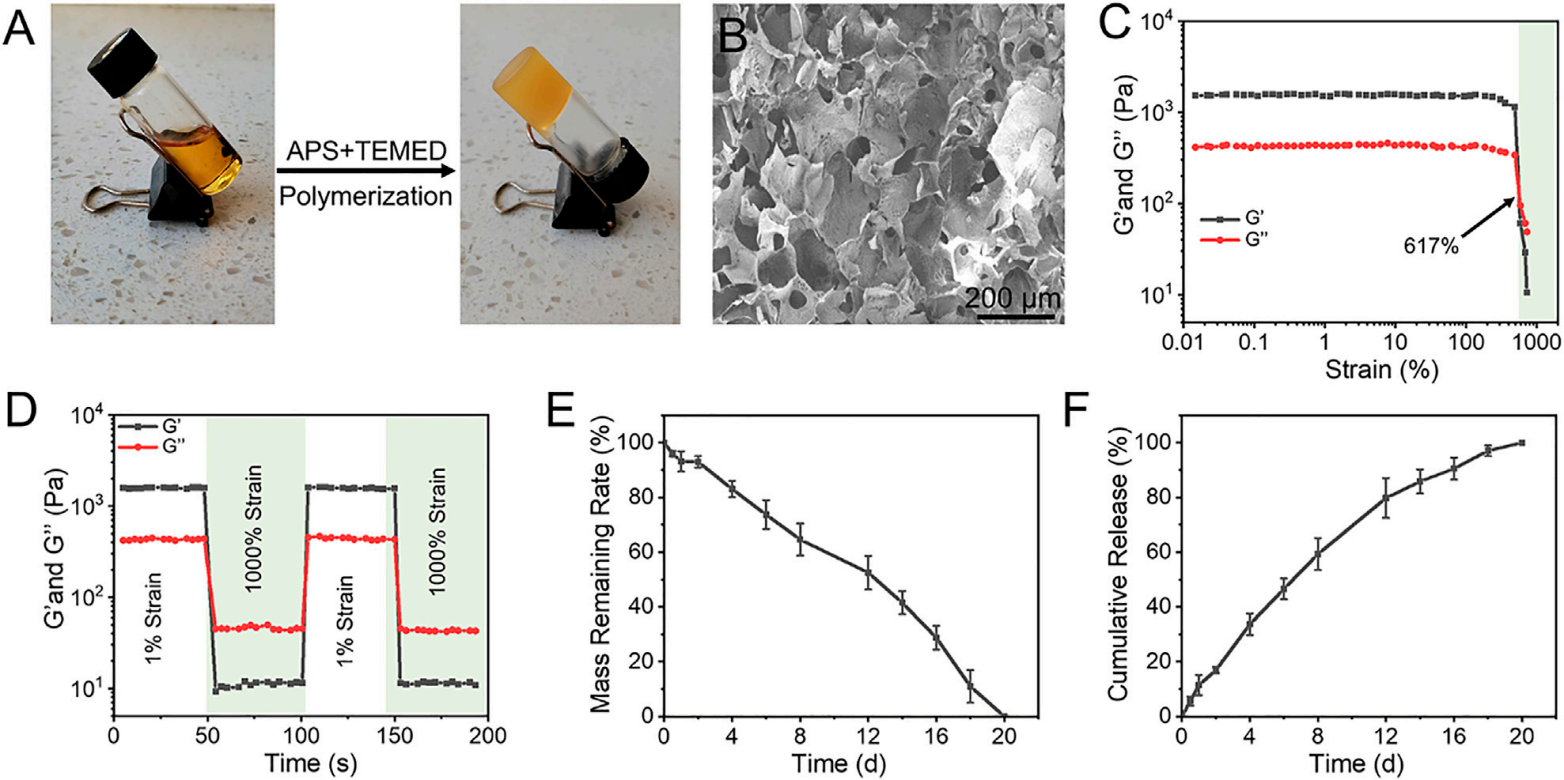
**(A)** The gross images of the sol–gel transition progress. The mixture of E-A complex and AM existed in a sol state at room temperature, and it completed gelation soon after adding APS and TEMED. **(B)** Morphologies of hydrogel observed by SEM. **(C)** Strain amplitude sweep test of hydrogel with constant frequency of 10 rad s^−1^ and varied strain. **(D)** G′ and G″ of hydrogel during two cycles between 1% and 1000% strain, each strain interval was kept as 50 s. **(E)** Degradation profile of the hydrogel in PBS solution at 37°C. **(F)** Releasing profiles of EGCG from the hydrogel in PBS solution at 37°C.

**FIGURE 2:**
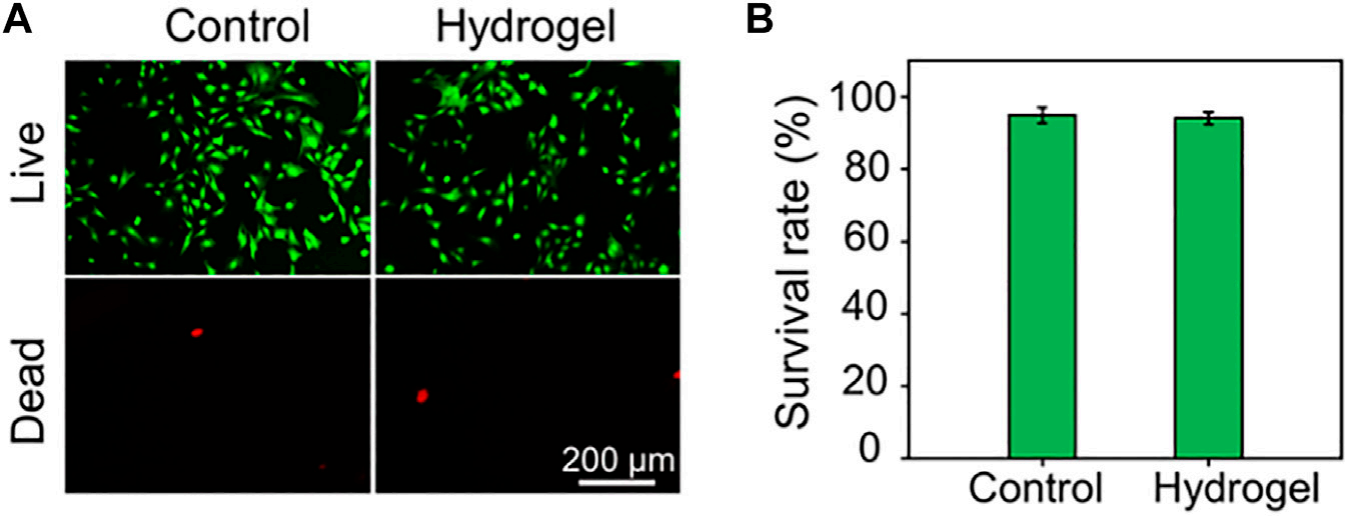
**(A)** Calcein AM/PI staining of rBMSCs. The green signal labels the live cells, whereas the red signal labels the dead cells. **(B)** Quantitative analysis of cell survival rate by Calcein AM/PI staining.

The catechol group in EGCG can form two reversible borate ester bonds with phenylboronic acid (PBA), which can be used as a dynamic crosslinking agent for constructing functional hydrogels (Huang et al., 2019; Zhang et al., 2019). Therefore, we assume that the dynamic crosslinking agent produced by APBA and EGCG can be used to synthetize functional hydrogels with desirous capacities by one-pot free radical polymerization with some water-soluble monomers (Zhao et al., 2021). As exhibited in Figure 1A, the mixture of the obtained E-A complex and AM aqueous solution existed in a sol state at room temperature, and completed sol–gel transition soon after adding APS and TEMED. In this study, the ratio of two for APBA to EGCG was used to prepare the pre-assembled complex. The formed E-A complex as dynamic crosslinker was used to directly copolymerize with AM in the presence of TEMED and APS to obtain the resulting hydrogel facilely. The images of SEM indicated that the resulting hydrogel showed regular interconnected porous structure, with a pore size of 100–200 μm (Figure 2B), which may give the hydrogel with sufficient permeability and provide space for nutrient transport and cell migration (Lu et al., 2019; Bai et al., 2020b; Li et al., 2021).

The representative strain sweep detection of the hydrogel showed that the values of Gʹ and G″ were almost constant under low strain change. With the further increase of strain, Gʹ and G″ intersected with at a strain of 617%, indicating that the hydrogel underwent the gel-sol transition process and then had a liquid-like rheological behavior (Figure 1C). Hydrogels constructed by boronate ester bonds continually experience dynamic reversible interactions between the reactants (boronic acid derivatives and catechols) and the products (boronate ester bonds), therefore, the hydrogels generally show excellent self-healing capacity. As exhibited in Figure 1D, rheology studies of the strain amplitude sweep were then conducted to observed the self-healing behavior of the hydrogel. The strain switching showed that the structure of the hydrogels was broken at high strain, and the original performance can be restored at high strain. Degradation behavior is a crucial performance for the biological application of hydrogels. The residual weight of hydrogels gradually decreased with the extension of incubation time in PBS, and they were completely degraded at 20 days (Figure 1E). With the gradual broken of the network and stable degradation, EGCG continuously released from the hydrogel in a 20-day observation period (Figure 1F). Considering the superior antioxidant effect of EGCG, the most abundant polyphenol compound in green tea, this sustained release profiles may endow the hydrogel with stable and long-term ROS capacity in the disease microenvironment.

### Biocompatibility and ROS-Scavenging capacity of hydrogel

Before biological applications, the biocompatibility of the hydrogel was evaluated by Calcein AM/PI staining. As depicted in Figure 2A, rBMSCs seeded in either the plate as Control group or in the hydrogel almost showed green stained living cells. The cell survival rates of rBMSCs in the Control group and Hydrogel group were 94.91 ± 2.09% and 94.06 ± 1.64%, respectively, with no statistical difference (Figure 2B). These results showed that the obtained hydrogel has good biocompatibility and potential to act as biomaterials.

**FIGURE 3:**
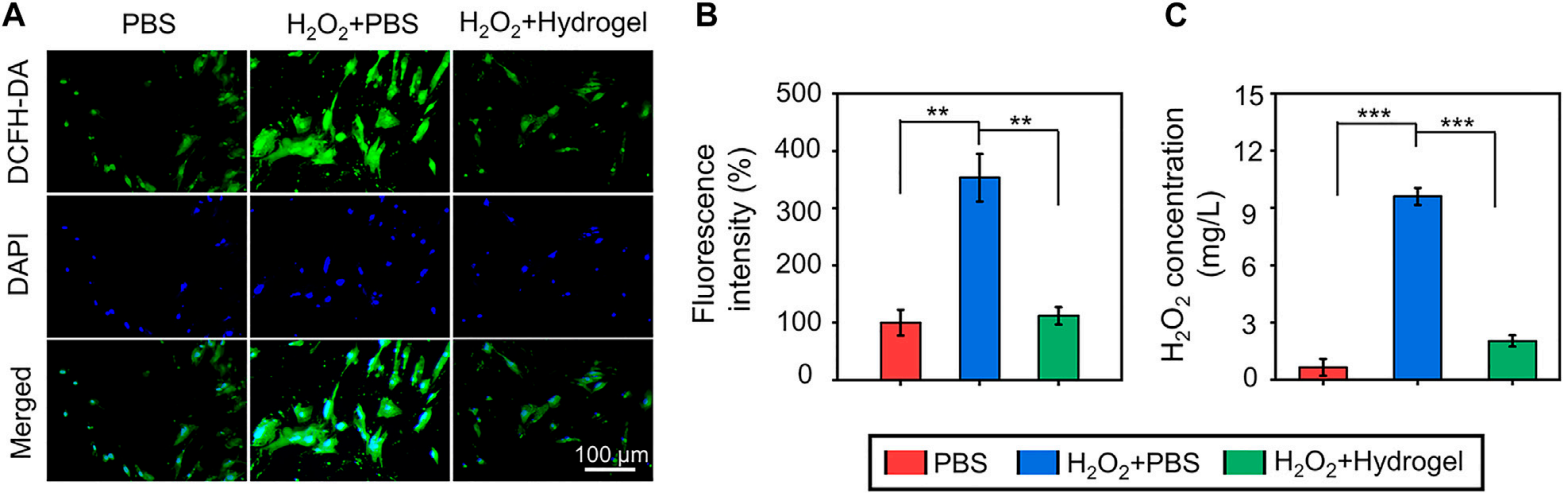
**(A)** The observation of ROS-scavenging ability by a ROS-specific probe (DCFH-DA). **(B)** The quantitative analysis of fluorescenceS intensity of ROS-scavenging. **(C)** The concentrations of H_2_O_2_ in culture medium evaluated by the typical Ti(SO_4_)_2_ colorimetric method (**p < .01 and ***p < .001).

Considering the accumulation of ROS in the osteoporotic microenvironment, cells were seeded on different substrates and incubated with 300 μM H_2_O_2_ to mimic the microenvironment *in vitro*. Subsequently, in order to observe anti-oxidant potential of rBMSCs on different substrates, intercellular ROS levels were detected through the DCFH-DA probe (Figure 3A). As exhibited in Figure 3B, the quantitative analysis of fluorescence intensity revealed that endogenous ROS level of cells cultured in H_2_O_2_ microenvironment was increased, and the introduction of hydrogel could alleviate the intercellular ROS accumulation. In addition, the concentrations of H_2_O_2_ in culture medium were further evaluated by the typical Ti(SO_4_)_2_ colorimetric method, and the results indicated that the EGCG-based hydrogel significantly decreased the H_2_O_2_ content in the medium compared with H_2_O_2_+PBS group (Figure 3C). As a cell delivery carrier, this hydrogel with superior ROS-scavenging capacity may provide an alternative solution for the survival and functional decline of delivered cells in the oxidative stress pathological microenvironment.

### ROS-scavenging hydrogel protects cell viability and osteoblastic differentiation under oxidative stress

**FIGURE 4:**
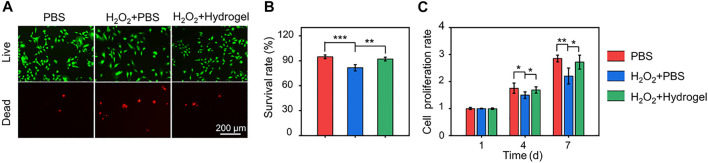
**(A)** Calcein AM/PI staining of rBMSCs for incubating 4 days. **(B)** Quantitative analysis of cell survival rates according to Calcein AM/PI staining. **(C)** Cell proliferation evaluated by CCK-8 assay for incubating 1, 4, and 7 days (*p < .05, **p < .01, and ***p < .001).

Under oxidative stress state induced by H_2_O_2_, cell viability decreases and differentiation ability is impaired. In this pathological microenvironment, the delivered cells, as seed cells of tissue engineering, are difficult to play a full role in repairing damaged tissues (Li et al., 2022a). Therefore, it is of great practical significance to develop cell delivery systems with the ability to scavenge ROS efficiently and continuously. In this study, EGCG-based hydrogel was developed to eliminate ROS continuously and its protective effect on cell activity under oxidative stress was observed by Calcein AM/PI staining (Figure 4A). The cell survival rates in the PBS, H_2_O_2_+PBS, and H_2_O_2_+Hydrogel groups were 94.77 ± 2.24%, 81.57 ± 3.70%, and 92.08 ± 2.22%, respectively (Figure 4B). In addition, the proliferation of rBMSCs on different substrates was detected by CCK-8 assay. As indicated in Figure 4C, rBMSCs incubated with hydrogel continued to proliferate for 7 days in this hostile microenvironment, and there was no significant difference compared with PBS group. The results demonstrated that compared with normal culture conditions (PBS group), the oxidative stress microenvironment induced by introducing H_2_O_2_ significantly inhibited cell survival and proliferation. However, by continuously eliminating ROS, the hydrogel may construct a suitable non-oxidative stress microenvironment for the survival and proliferation of rBMSCs, thus restoring cell activity.

**FIGURE 5:**
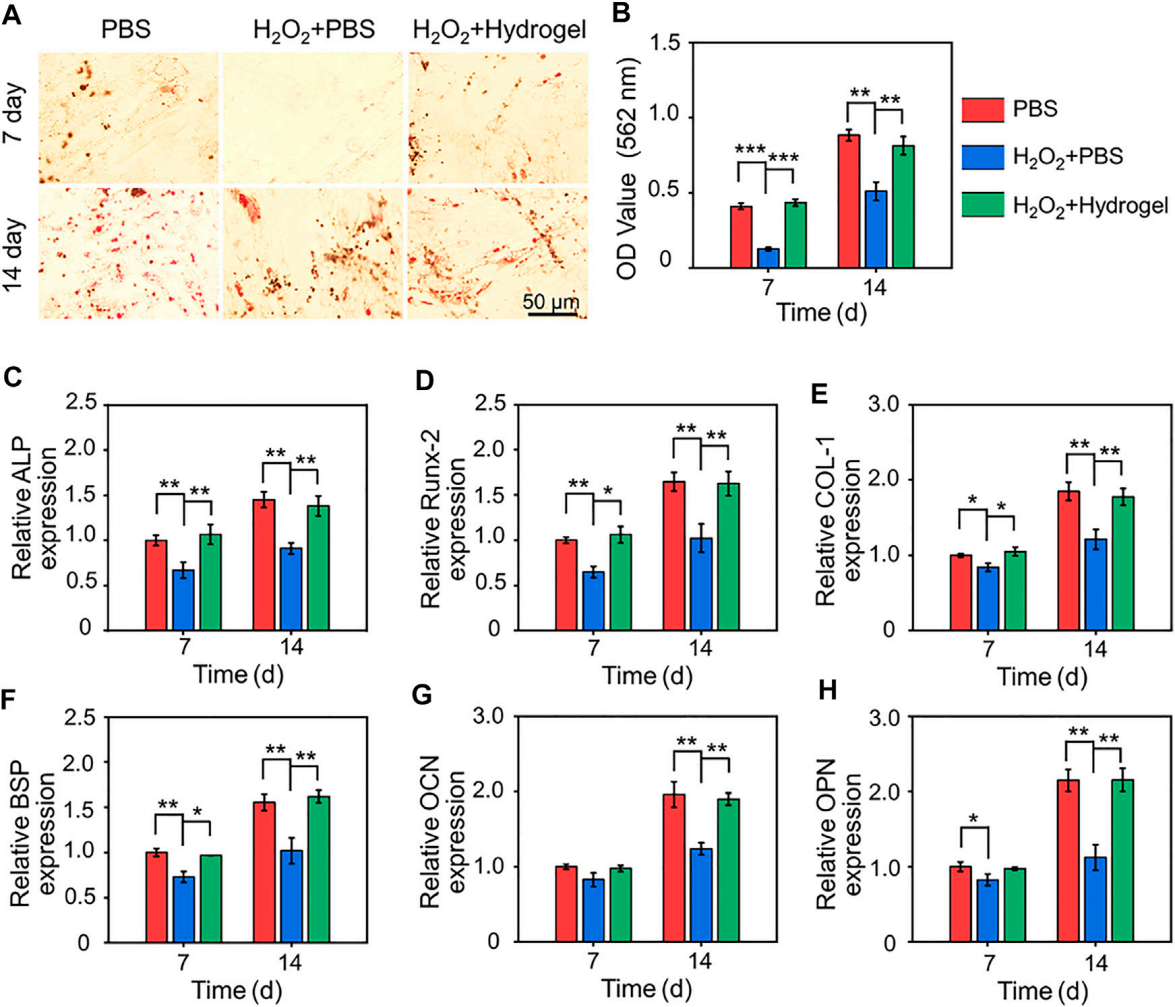
**(A)** Gross images of alizarin red staining after 7 and 14 days of cell culture. **(B)** Semi-quantitative analysis of the deposition of calcium nodules by alizarin red staining. **(C–H)** Expression of osteogenesis-related genes in different groups (*p < .05, **p < .01, and ***p < .001).

As seed cells, the osteogenic differentiation potential of BMSCs delivered to bone defects is crucial for bone healing. Therefore, the osteoblastic differentiation potential of rBMSCs on different substrates were evaluated by alizarin red staining of mineralized nodules and PCR analysis of osteogenesis related genes. As showed in Figure 5A, obvious red stained mineralized nodules were observed in PBS group and H_2_O_2_+Hydrogel group on the 7th and 14th days of osteogenic differentiation induction. Further quantitative analysis showed that the mineralization of rBMSCs in H_2_O_2_+Hydrogel group was similar to that of PBS group, which was significantly higher than that of H_2_O_2_+PBS group (Figure 5B). In addition, we also investigated that the expression of the osteogenesis-related genes (including Runx2, ALP, COL-1, and BSP) in H_2_O_2_+Hydrogel group was significantly higher than that of H_2_O_2_+PBS group on the 7th and 14th days (Figures 5C–F). However, the classic markers of late osteogenesis, OPN and OCN showed significant difference between H_2_O_2_+PBS group and H_2_O_2_+Hydrogel group until the 14th day (Figures 5G, H). To sum up, the presence of H_2_O_2_ will inhibit the osteogenic differentiation ability of rBMSCs, which is specifically manifested by the reduction of calcium nodule deposition and the downregulation of osteogenic markers, consistent with previous studies (Chen M. et al., 2022; Shen et al., 2022). However, the introduction of EGCG-based hydrogel with ROS-scavenging capacity can reshape the oxidative stress microenvironment in order to facilitate the osteogenic differentiation of rBMSCs. Thus, it paved the way for the subsequent application of *in vivo* delivery of stem cells to promote prosthetic interface osteointegration in osteoporosis.

### 3D-printed bioactive prosthesis interface reshapes osteogenic microenvironment of osteoporosis

**FIGURE 6:**
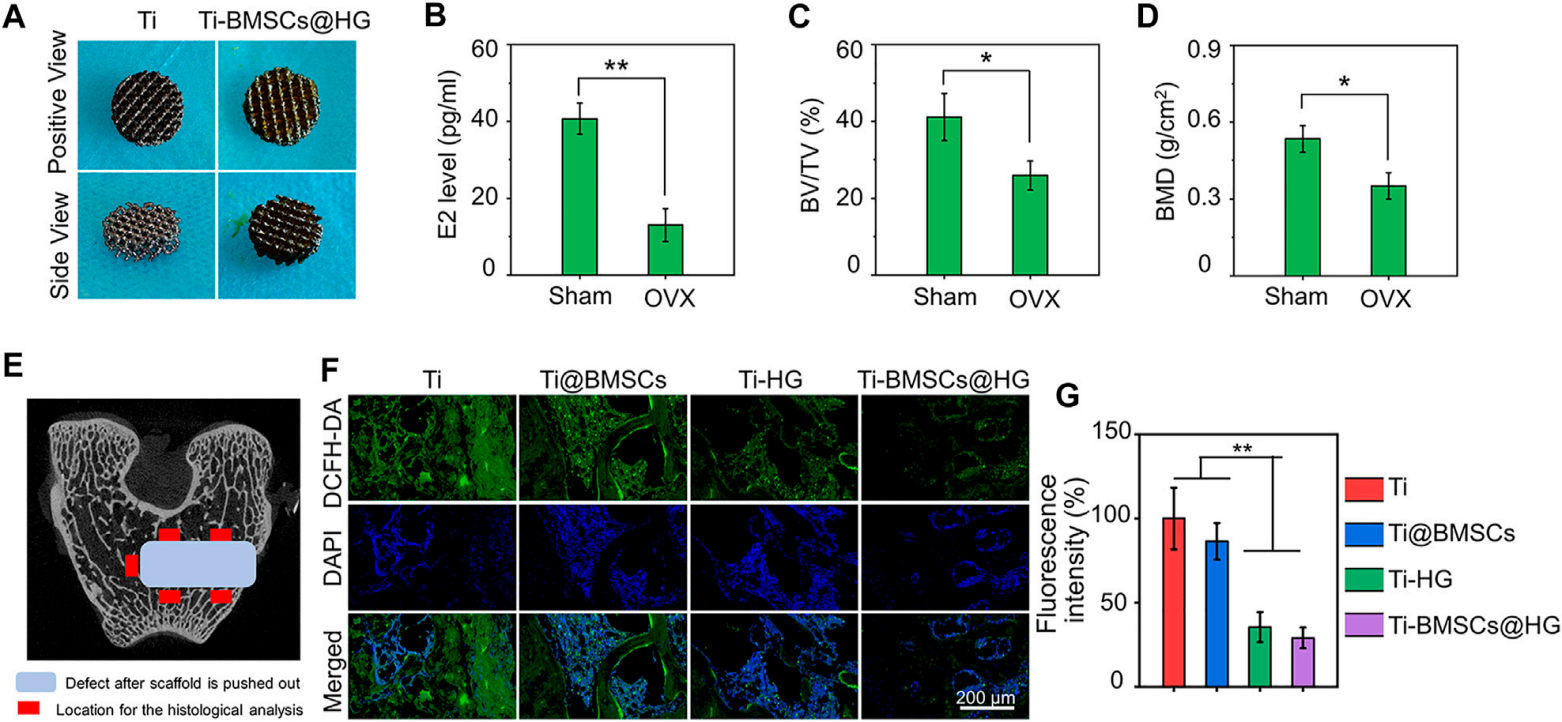
**(A)** Gross images of 3D printed porous titanium alloy prosthesis and rBMSCs-loaded hydrogel filled porous prosthesis. **(B)** Serum estrogen (E2) concentrations of rabbit model 8 months after OVX. **(C)** Quantitative analysis of BV/TV 8 months after OVX by Micro-CT scanning. **(D)** Quantitative analysis of BMD 8 months after OVX by Micro-CT scanning. **(E)** The schematic picture of the location for the histological analysis. **(F)** ROS-scavenging around the interface 2 weeks after prosthesis implantation was detected by a ROS probe (DCFD-DA). **(G)** Quantification of the fluorescence intensity of DCFD-DA in bone samples (*p < .05 and **p < .01).

3D-printed technology is a new strategy for the preparation of orthopedic prostheses, which can manufacture customized implants with complex structures layer by layer with high precision to meet the requirements of complex osteopathy in different situations (Zhao et al., 2017; Lee et al., 2022). As shown in Figure 6A (left), porous titanium alloy prosthesis was successfully manufactured through 3D-printed technology. The porosity of the prepared scaffolds was confirmed by Micro-CT, and the pore size distribution was analyzed by SEM. The porosity of the scaffolds was 70.1%, which was in line with the original design (70%). And then ImageJ analysis of the SEM pictures indicated that the pore size of the obtained scaffolds was 798.8 μm. Previous studies have shown that 3D printed porous titanium alloy prosthesis with these parameters could improve the initial implant stability, bone ingrowth capacity, and the friction coefficient between the bone and scaffolds, thus reducing micromotion and promoting osseointegration *in vivo* (Bai et al., 2020a; Bai et al., 2020c). Owing to this 3D porous interconnected structure, the rBMSCs-loaded hydrogel was filled into the micropores of prosthesis to construct a 3D-printed bioactive prosthesis interface for transplantation (Figure 6A right). In addition, as displayed in Figures 6B–D, compared with the sham operation group, the serum estrogen level of rabbits in the OVX group greatly decreased, and the values of BV/TV and BMD in distal femur diminished significantly, which was consistent with the classical osteoporosis model in previous studies (Cui et al., 2021; Sadat-Ali et al., 2021).

**FIGURE 7:**
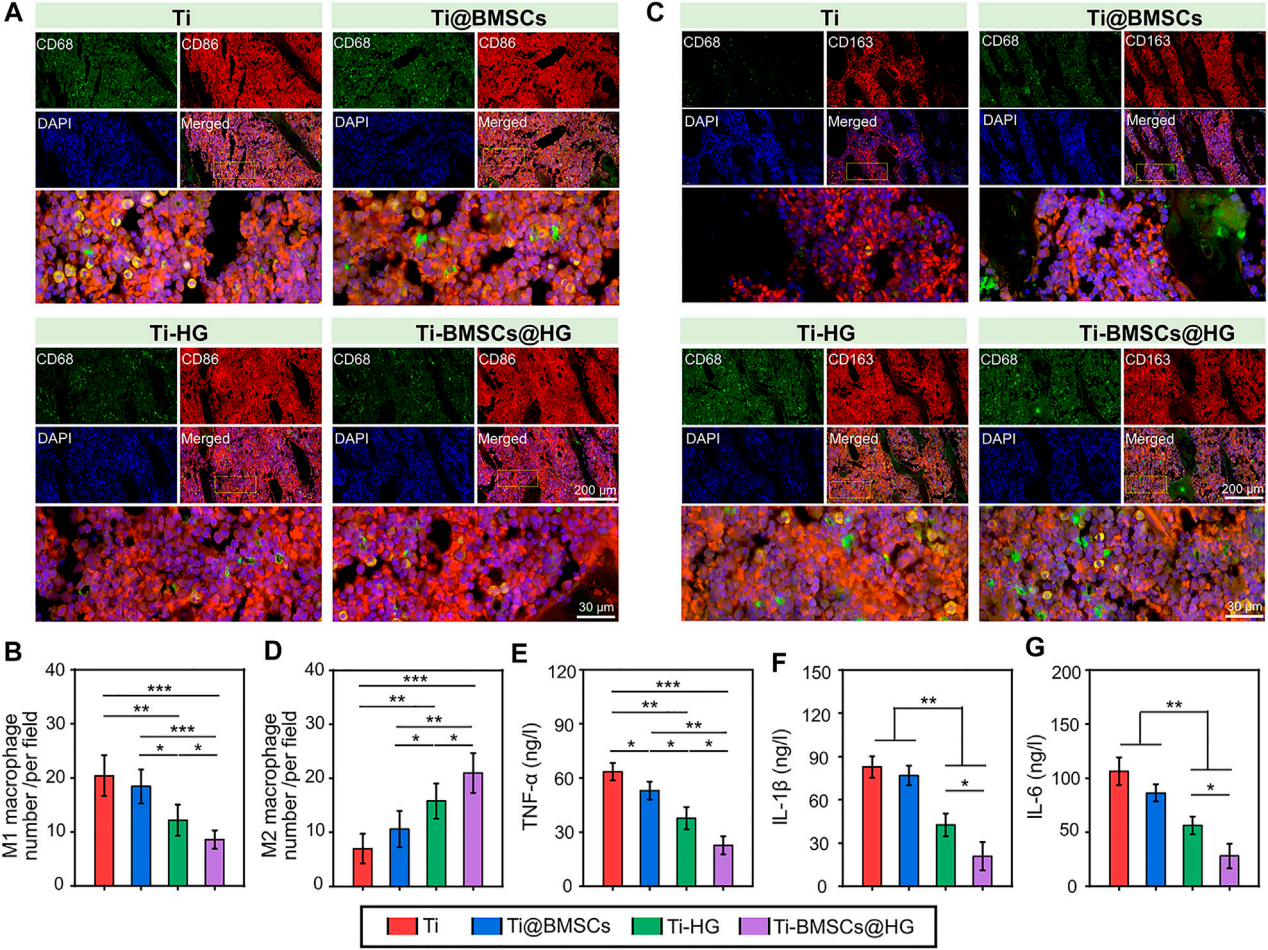
**(A)** Representative immunofluorescence staining images of CD68 plus CD86 to label M1 phenotype macrophages after implantation for 2 weeks. **(B)** Quantitative analysis of the number of M1 phenotype macrophages. **(C)** Representative immunofluorescence staining images of CD68 plus CD163 to label M2 phenotype macrophages after implantation for 2 weeks. **(D)** Quantitative analysis of the number of M2 phenotype macrophages. **(E–G)** The concentrations of TNF-α, IL-1β, and IL-6 in bone marrow blood around the prosthesis interfaces (*p < .05, **p < .01, and ***p < .001).

When different prostheses were transplanted to the lateral condyle of the distal femur of osteoporotic rabbits for 2 weeks, we collected bone tissues and analyzed their regulatory effects on the local microenvironment. As shown in Figure 6E, the schematic picture of the location for the histological analysis displayed that we select five visual fields of bone around the implant for analysis and calculate the average value as the parameter of this sample. The ROS probe (DCFH-DA) was used to evaluate the accumulation of ROS around the prosthesis interfaces (Figure 6F). As illustrated in Figure 6G, the quantitative analysis showed that the introduction of hydrogel into the micropores of prosthesis could significantly reduce the fluorescence intensity of DCFH-DA, that is, the level of ROS in Ti-HG group and Ti-BMSCs@HG group were significantly lower than that in Ti group and Ti@BMSCs group (*p* < .01). Furthermore, to study the macrophages polarization around different prosthesis interfaces, immunofluorescence staining of CD68 plus CD86 (labeling M1 phenotype macrophages) and CD68 plus CD163 (labeling M2 phenotype macrophages) were conducted. As depicted in Figures 7A–D, in the Ti-HG group and Ti-BMSCs@HG group where the accumulated ROS was alleviated, the number of pro-inflammatory M1 macrophages was significantly reduced, while the number of M2 regenerative macrophages was significantly increased, compared with the Ti group and Ti@BMSCs group. Subsequently, various inflammatory cytokines in bone marrow blood around the different prosthesis interfaces were detected to evaluate the local inflammatory status. As exhibited in Figures 7E–G, among the groups, the concentrations of TNF-α, IL-1β, and IL-6 decreased most significantly in Ti-BMSCs@HG group.

Persistent oxidative stress is one of important factors of macrophages polarization disorder (tendency to pro-inflammatory M1 phenotype) and continuous chronic low-grade inflammation in osteoporotic microenvironment, leading to limited bone regeneration and osteointegration (Hu et al., 2021; Chen Q. et al., 2022). Therefore, regulating the local immune environment affected by excessive ROS is considered as a new strategy to restore the internal balance of bone metabolism in osteoporosis. Say concretely, in the ROS accumulation and persistent inflammatory microenvironment, EGCG-based hydrogel could alleviate inflammatory responses in the early phase through the sustained release of EGCG and accelerate the transition from the inflammation phase to the proliferation phase by promoting the transformation of macrophages from pro-inflammatory M1 to reparative M2 phenotype. In addition, after EGCG-based hydrogel application, the content of pro-inflammatory chemokines (IL-1β and IL-6) was significantly reduced, the content of anti-inflammatory cytokines (IL-4 and IL-10) and reparative growth factors (TGF-β1 and VEGF) were also enhanced, indicating the effectively promoted transformation effect on macrophages from inflammatory M1 to reparative M2 phenotype (Zhao et al., 2021c). In this study, our prepared EGCG-based hydrogel alleviated ROS accumulation in osteoporotic microenvironment, thus reducing the number of pro-inflammatory M1 macrophages and increasing the number of M2 regenerative macrophages. It is worth mentioning that the effect of Ti-BMSCs@HG group on inducing macrophages to polarize from M1 to M2 is more obvious than that of Ti-HG group, which may be attributed to the immune regulation of loaded rBMSCs in a relieved osteoporosis microenvironment (Liu C. et al., 2022; Shao et al., 2022).

**FIGURE 8:**
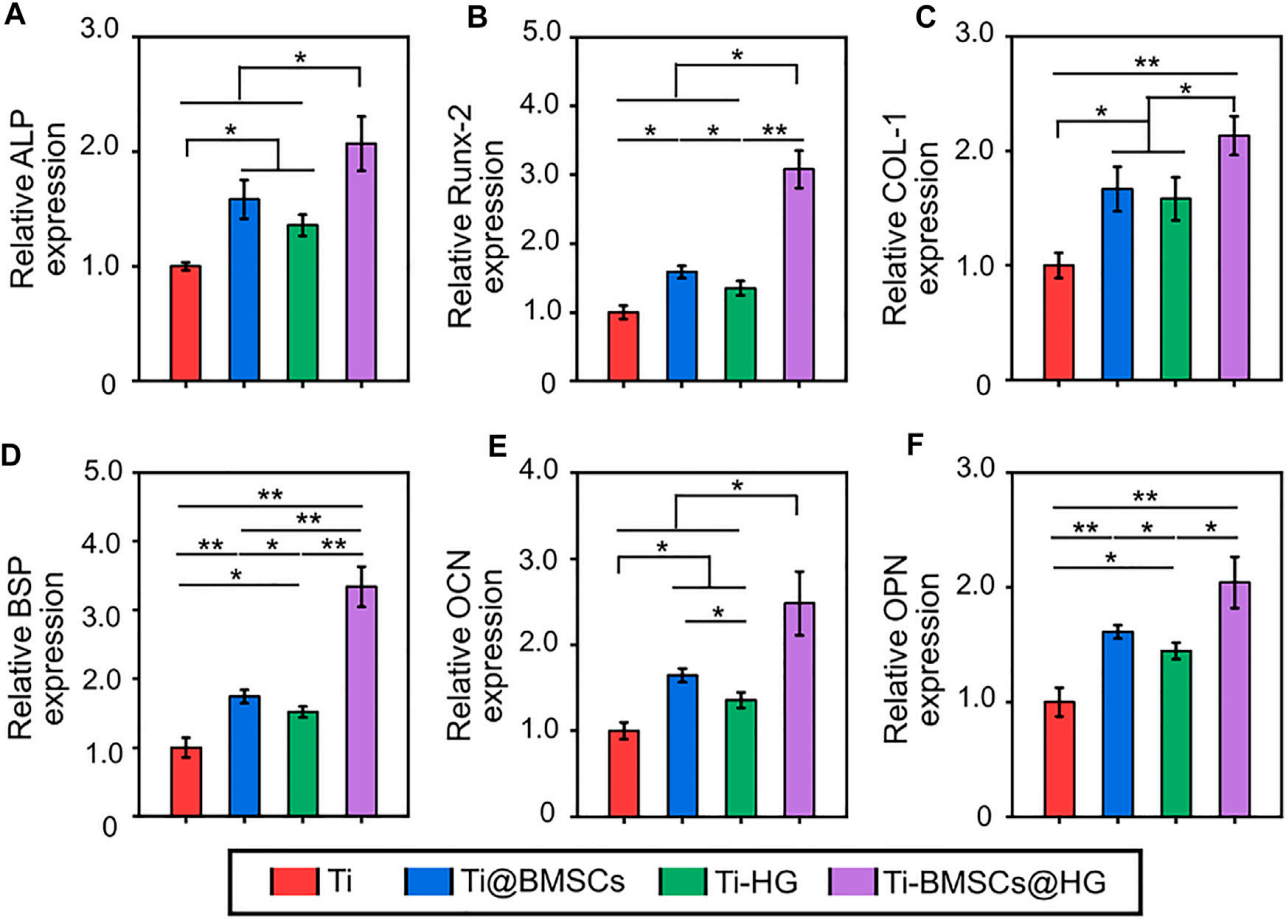
Expression of osteogenesis-related genes in bone tissues in the pores of porous prostheses, including **(A)** ALP, **(B)** Runx-2, **(C)** COL-1, **(D)** BSP, **(E)** OPN, and **(F)** OCN (*p < .05 and **p < .01).

Obviously, compared with direct delivery of stem cells to bone defects (Ti@BMSCs group), encapsulation of rBMSCs by EGCG-based hydrogel as a protective carrier (Ti-BMSCs@HG group) can significantly improve the expression of various osteogenic related genes, including Runx2, ALP, COL-1, BSP, OPN, and OCN, in the microenvironment around the prosthesis interfaces (Figures 8A–F). In general, the EGCG-based hydrogel, as a protective carrier for delivering stem cells, combined with 3D-printed porous prosthesis, can effectively remove excessive ROS, induce macrophages to polarize from M1 to M2 phenotype, decrease the expression of proinflammatory cytokines in the osteoporosis microenvironment, thus up-regulating the osteogenic markers and reshaping the osteogenic microenvironment to create favorable conditions for the bone integration of prosthesis in osteoporosis.

### 3D-printed bioactive prosthesis interface enhances osteointegration in osteoporosis

In osteoporosis, estrogen deficiency will lead to the rapid accumulation of ROS in the bone marrow microenvironment. The increasing H_2_O_2_ level would suppress the differentiation of BMSCs and seriously hinder the early bone regeneration and prosthesis interface osseointegration, leading to delayed bone healing and increased risk of secondary surgery (Ensrud, 2021). In addition, the number of endogenous BMSCs in osteoporotic bone defects is usually insufficient. These reasons seriously hinder the osteointegration of implants in osteoporosis, leading to an increased risk of complications such as prosthesis loosening, displacement and periprosthetic fractures after surgery (Ensrud, 2021; Lu et al., 2022). Therefore, inhibiting the excessive accumulation of ROS can significantly improve the osteogenic microenvironment, thereby promoting osteogenic differentiation of BMSCs and accelerating bone regeneration and prosthetic osteointegration under osteoporosis (Shen et al., 2021; Chen Q. et al., 2022).

**FIGURE 9:**
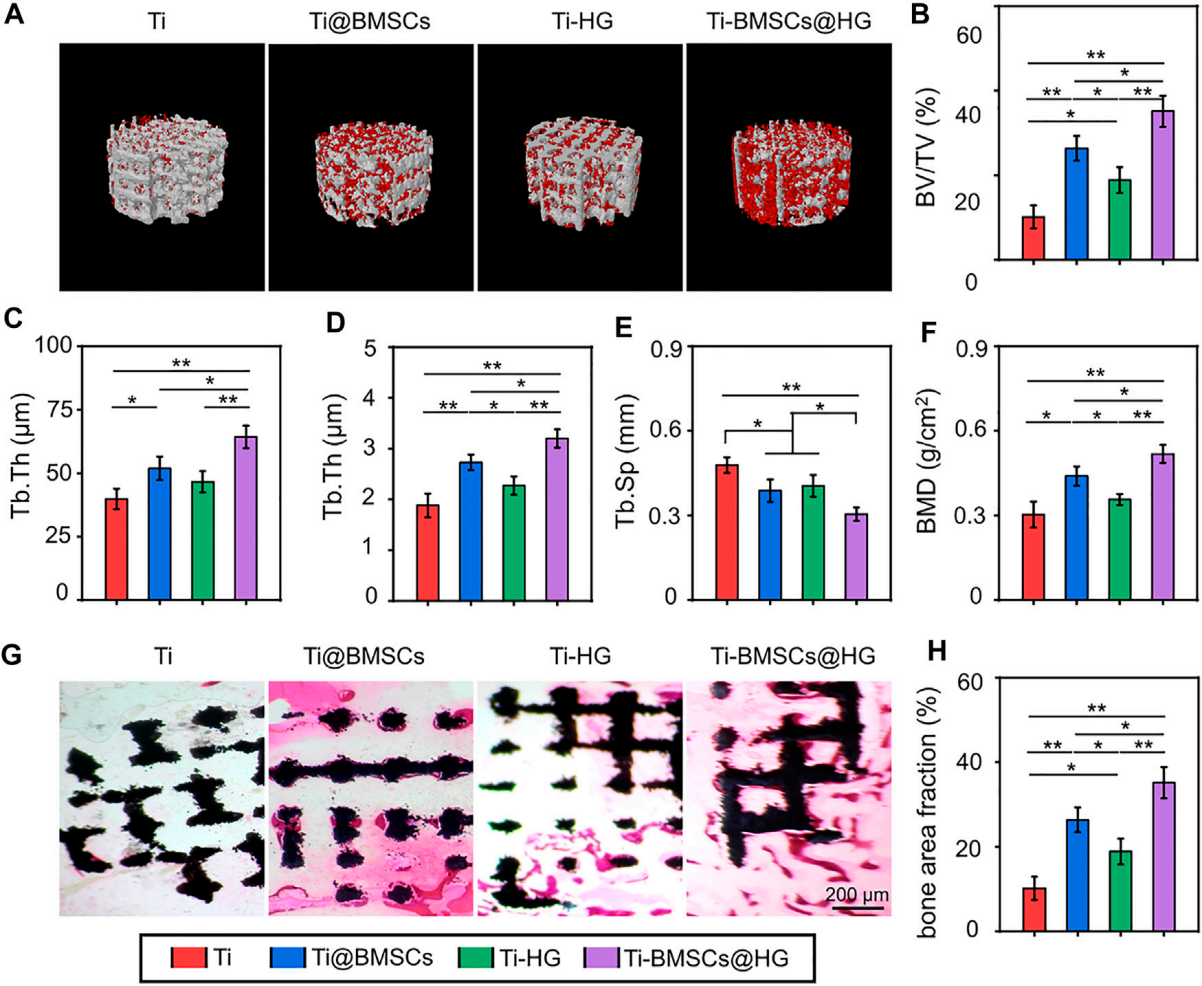
**(A)** 3D reconstruction images of porous titanium alloy prosthesis (white) and new bone formation (red). **(B–F)** Quantitative analysis of bone morphological parameters, including BV/TV, Tb.Th, Tb.N, Tb.Sp, and BMD according to Micro-CT at 10 weeks after implantation. **(G)** Representative histological images of H&E staining (The black indicated the porous titanium alloy prosthesis and the red areas indicated the new formation bone tissues). **(H)** Quantitative analysis bone area fraction by H&E staining (*p < .05 and **p < .01).

As listed in Figure 9A, 10 weeks after different prostheses implantation, the bone formation around the prosthesis interfaces was assessed by Micro-CT and visually presented by 3D reconstruction. The representative images indicated that the Ti-BMSCs@HG group showed a better bone-implant integration, with the most obvious new bone formation. Subsequently, the corresponding quantity parameters showed similar results, indicating that the Ti-BMSCs@HG group possessed the highest results of BV/TV, Tb.Th, Tb.N, and BMD, as well as the lowest results of Tb.Sp among the four groups (Figures 9B–F). Sufficient osseointegration between implant and host bone can reduce the occurrence of a series of prosthetic complications after joint replacement. In order to observe the bone growth in porous titanium alloy prosthesis and its integration with surrounding bone tissues, we prepared hard tissue sections for histological observation. As displayed in Figure 9G, representative H&E staining images demonstrated that regenerated bone tissues almost surrounded the surface of the porous prosthesis, and even fully grew into the deep pores. Quantitative analysis showed that the proportion of bone area in the prosthesis interfaces in Ti, Ti-@BMSCs, Ti-HG, and Ti-BMSCs@HG groups were 10.17 ± 4.07%, 24.07 ± 2.23%, 18.66 ± 2.35%, and 31.63 ± 3.29%, respectively, indicating the Ti-BMSCs@HG group obtained the best osteointegration result among the four groups (Figure 9H).

## Conclusion

In this study, an EGCG-based ROS-scavenging hydrogel is engineered to be a delivery vehicle of BMSCs to enhance 3D-printed porous titanium alloy prosthesis osseointegration in osteoporosis. The resulting hydrogel remolds the harsh osteogenic microenvironment of osteoporosis by removing ROS and protects implanted cells from ROS-mediated death and osteogenesis inhibition. As a cell delivery carrier, hydrogel loaded BMSCs were combined with porous prosthesis to construct a novel 3D-printed bioactive prosthesis interface. In osteoporosis, the 3D-printed bioactive prosthesis interface can effectively remove excessive ROS, induce macrophages to polarize from M1 to M2 phenotype, decrease the expression of proinflammatory cytokines, thus up-regulating the osteogenic markers and reshaping the osteogenic microenvironment to create favorable conditions for the osteointegration. This work provides a strategy for solving the challenge of transplanted stem cells cannot fully function in the impaired microenvironment, and enhancing prosthetic osteointegration in osteoporosis.

## Data Availability

The original contributions presented in the study are included in the article/Supplementary material, further inquiries can be directed to the corresponding authors.
